# "Conservative" Obstetrics

**Published:** 1902-04-26

**Authors:** 


### "CONSERVATIVE" OBSTETRICS.

During recent years a strong feeling has manifested itself in favour of so-called conservative methods in dealing with obstetric difficulties, the word "conservative" having reference more particularly to the child. Thus we have witnessed the increased employment of such measures as symphysiotomy, Cæsarean section, and induction of premature labour, and the results have been good, and even brilliant. In this, however, as in almost everything, the pendulum swings. It is already being said that with equal care and equal trouble in maintaining asepsis internal operations such as turning and craniotomy and cranioclasis have shown such an improvement in their (maternal) results as to reinstate them in their old position. Much depends on point of view. So far as the child is concerned, of course, the conservative methods have everything in their favour. But there are plenty of men who consider that their first consideration should be the safety of their patient, *i.e.* of the mother, the life of the unborn child being regarded as quite a secondary consideration. At a recent meeting of the Eastern Medical Society of New York Dr. S. Marx held that in a case of labour all one's energies should be concentrated on one purpose—to injure the mother as little as possible. Viewed from this standpoint, he said, perforators, craniotomes, cranioclasts, etc., were conservative instruments, and not relics of barbarism, as some would lead one to think. He further held that a clear recognition of the indications for the use of the destructive instruments was of the greatest importance, since their purpose is best served by their early application. In these cases, he said, the condition of the badly maimed or dying fœtus must never be seriously taken into consideration. In the same discussion Dr. Barshy, speaking as a general practitioner working in a tenement district, spoke of the limitations imposed by the surroundings of the patient, and (in regard to symphysiotomy) by the necessity of insuring a rapid restoration of capacity for doing house-work. The only procedures which he had found applicable in his practice were version, forceps, and craniotomy. So the pendulum swings. And we have no doubt that a careful and aseptic practitioner who limited himself to a systematic and early recourse to one or other of these procedures would do very well—for the mothers. The question is, Is it right?

